# Discrimination Ability of Assessors in Check-All-That-Apply Tests: Method and Product Development

**DOI:** 10.3390/foods10051123

**Published:** 2021-05-19

**Authors:** Attila Gere, Dávid Bajusz, Barbara Biró, Anita Rácz

**Affiliations:** 1 Department of Postharvest, Supply Chain, Commerce and Sensory Science, Institute of Food Science and Technology, Hungarian University of Agriculture and Life Sciences, Villányi út 29-43, H-1118 Budapest, Hungary; barbarabirophd@gmail.com; 2 Medicinal Chemistry Research Group, Research Centre for Natural Sciences, Magyar Tudósok krt. 2, H-1117 Budapest, Hungary; bajusz.david@ttk.mta.hu; 3 Plasma Chemistry Research Group, Research Centre for Natural Sciences, Magyar Tudósok krt. 2, H-1117 Budapest, Hungary; racz.anita@ttk.mta.hu

**Keywords:** panelist performance, discrimination ability, CATA, product development, binary similarity

## Abstract

Binary similarity measures have been used in several research fields, but their application in sensory data analysis is limited as of yet. Since check-all-that-apply (CATA) data consist of binary answers from the participants, binary similarity measures seem to be a natural choice for their evaluation. This work aims to define the discrimination ability of CATA participants by calculating the consensus values of 44 binary similarity measures. The proposed methodology consists of three steps: (i) calculating the binary similarity values of the assessors, sample pair-wise; (ii) clustering participants into good and poor discriminators based on their binary similarity values; (iii) performing correspondence analysis on the CATA data of the two clusters. Results of three case studies are presented, highlighting that a simple clustering based on the computed binary similarity measures results in higher quality correspondence analysis with more significant attributes, as well as better sample discrimination (even according to overall liking).

## 1. Introduction

In food sensory analysis, check-all-that-apply (CATA) experiments show an increasing tendency among practitioners. Its high popularity is due to the simplicity, effectiveness, and fast execution, as well as the many different applications [1]. Application fields of CATA is not restricted to consumer assessors, researchers used the method with semi-trained assessors as well [2]. 

During a CATA experiment, a list of sensory attributes is presented to the assessors, from which they choose only those they identify in the given sample [3]. The final data table of such an analysis contains the products and assessors in its rows and the sensory attributes in the columns. The table is binary, where 1 denotes if the assessor identified the attribute in the sample while 0 means that the attribute was not perceived by the assessor. The most obvious way to analyze such data is to create contingency tables which show how many times a given attribute was chosen. 

CATA data analysis is a widely researched area; however, one of the most known methods is graphical correspondence analysis (CA). CA might be considered as a generalized principal component analysis (PCA) for ordinal data [4]. Its popularity is due to the easy interpretability of the results and the high similarity with the well-known biplots of PCA. Attribute-wise differences among samples are analyzed by Cochran’s test, while a modified penalty analysis is widely used to define attributes with the greatest effect on the overall liking of the products [5,6].

It is also important to evaluate the panel participating in a CATA experiment. Reproducibility was investigated first among the performance metrics and within-assessor reproducibility index was introduced [7]. Recent papers on CATA data analysis focus on the clustering of subjects in CATA experiments. Since CATA participants are usually consumers, monitoring their performance is rather difficult. It is hypothesized that clustering participants based on their evaluations (e.g., their perceptions of the products) enables the researchers to conduct CAs on the different consumer groups. In order to get a dataset acceptable for cluster analysis, the CATATIS method has been introduced recently, which yields a weighted average dataset that cross-tabulates the products and the attributes. The original dataset is decomposed into assessor datasets, and during CATATIS, the group average dataset closest to the original dataset is identified to enable the definition of assessor weights. In the second step, these weights are used to create clusters of assessors based on their distance from the panel average, this is termed as the CLUSCATA method [8].

Further analysis of the agreement among the participants has also been discussed. The authors of CLUSCATA and CATATIS introduced an overall index of agreement among participants based on the cosine between two matrices associated with two respondents in a Euclidean space [9].

From the above-mentioned developments in CATA data analysis, it can be seen that there is a lack of methods aiming to describe the discrimination ability of consumer panels. There are multiple methods which are used to assess the discrimination ability of trained panels conducting descriptive sensory methods (e.g., F-plot [10] or MAM-CAP [11]).

In the present study, the application of binary similarity indices will be introduced and discussed as another tool for CATA data analysis. Binary similarity indices are widely used in various scientific fields, especially in chemometrics and cheminformatics to compare binary vectors [12,13]. Although their sensometric application was limited so far, we have recently highlighted their applicability for metabolomics data [14]. The most comprehensive collection of these metrics was published by Todeschini and co-workers [15]. Their work includes 44 different similarity metrics that were introduced by researchers of diverse fields.

The presented study aims to introduce a new approach for CATA data analysis which enables the researchers to:

- Assess the discrimination ability of the assessors;
- Create consumer clusters based on the level of discrimination of the assessors.

This is based on the application of binary similarity metrics (as published in our recent work and available at https://github.com/davidbajusz/fpkit, accessed on 18 May 2021) to three case studies of CATA evaluation scenarios. The python code used for the presented calculations, along with an example input is published open source in the same GitHub repository to allow for easy reproducibility of our results and the application of the presented approach to other CATA datasets.

## 2. Materials and Methods

### 2.1. Datasets

#### 2.1.1. Cricket Enriched Biscuit Dataset

Oat biscuit samples were prepared using house cricket (*Acheta domesticus*) powder, oat, and buckwheat flour. Cricket powder was shipped from Thailand (JR Unique Foods Ltd.; Udon Thani, Thailand), while oat and buckwheat flour were purchased from Hungarian producers (Első Pesti Malom-és Sütőipari Zrt. and Bonetta Bt; Budapest, Hungary). Other ingredients of the doughs were lactose free butter and sour cream, baking powder and salt. Samples contained 0% (Ctrl), 5% (CP5), 10% (CP10), and 15% (CP15) cricket powder. Sixty-seven consumers evaluated the prepared four samples using 38 CATA attributes (Table 1) in a one-week session at the Sensory Evaluation Laboratory of the Hungarian University of Agriculture and Life Sciences, Institute of Food Science and Technology. Participants received written information about the possible risks and allergens of the tested products and signed a consent form that they were aware of these risks and allergens, as well as that their blind answers would be used only for research purposes. The study was conducted according to the Guidelines of the Hungarian University of Agriculture and Life Sciences and was approved by the Ethical Committee of the Institute of Food Science and Technology.

#### 2.1.2. Gluten Free Brown Rice Biscuits Enriched with Apple Pomace and Flax Seeds Dataset

Biscuit samples were prepared using brown rice flour, whole flax seeds, and dried, finely ground apple pomace. Brown rice flour and flax seeds were purchased from Hungarian producers (Riceland-Magyarország Kft. and Dénes Natura Kft; Lajosmizse, Hungary). Apple pomace was made from Idared apples, at the Department of Food Preservation at Hungarian University of Agriculture and Life Sciences, Institute of Food Science and Technology. Other ingredients of the doughs were margarine, icing sugar, and vanilla sugar. Samples contained 0% (AP0), 2.5% (AP2.5), 5% (AP5), and 10% (AP10) ground apple pomace. Sixty consumers evaluated the prepared four samples using 34 CATA attributes (Table 1) in a one-week session at the Sensory Evaluation Laboratory of Hungarian University of Agriculture and Life Sciences, Institute of Food Science and Technology. Participants received written information about the possible risks and allergens of the tested products and signed a consent form that they were aware of these risks and allergens, as well as that their blind answers would be used only for research purposes. The study was conducted according to the Guidelines of Hungarian University of Agriculture and Life Sciences and was approved by the Ethical Committee of the Institute of Food Science and Technology.

#### 2.1.3. Strawberry Dataset

Strawberry varieties were analyzed by 117 consumers in a supermarket in Montevideo, Uruguay. Consumers rated 16 CATA attributes (Table 1) and overall liking. The dataset is widely used to illustrate CATA data analysis methods and was originally published by Ares and Jaeger (2013). Cochran’s Q test and multivariate data analysis proved that most attributes are discriminant [1]. Additionally, a more recent study identified a rather poor agreement among the subjects [8].

### 2.2. Data Analysis

#### 2.2.1. Binary Similarity Measures

The CATA experiments provide a list of sensory attributes, in which the presence of an attribute in a sample is denoted by “1” by the assessors. In this way, a long vector with the binary coded absence or presence of the attributes in the sample is given for each sample (row). The samples can be compared based on their binary vectors using a 2 × 2 table, which is presented in Table 2. The frequencies of 1-1, 0-0, and 0-1/1-0 pairs are calculated in the 2 × 2 table, based on the attributes in the input matrix. The four cases are the following: (a) 1-1 if the attribute is selected in both samples, (b) 1-0 if the attribute is selected in the first sample out of the two, (c) 0-1 if the attribute is selected in the second sample out of the two and (d) 0-0 if the attribute is selected in none of the compared two samples. Thus, four parameters (a, b, c, d) are calculated based on a pairwise comparison of the binary vectors. The additional parameter is the length of the binary vectors, which is marked by p.

The mentioned five parameters are the basis of the different equations of the binary similarity metrics. These are summarized in the work of Todeschini et al. [15], as well as our earlier work [14], and a few examples are given here: Tanimoto coefficient (JT)**,** Simple matching coefficient (SM)**,** Driver-Kroeber or cosine coefficient (DK).

JT = a/(a + b + c)(1)

SM = (a + d)/p(2)

DK = a/√((a + b)(a + c))(3)

Similarity and distance metrics can be transformed into each other in the following way:

Similarity = 1/(1 + Distance)(4)

Similarity metrics often, but not always, fall in the range between 0 and 1. For those metrics, which are in a different range, Todeschini et al. have proposed a simple scaling method to transform them to the [0, 1] range:

Similarity_scaled_ = (Similarity + α)/β (5)

where the α and β parameters are individually determined for each metric. In this work, the scaled versions of the 44 similarity metrics were calculated with the recently published FPKit Python package [13], which is freely available at https://github.com/davidbajusz/fpkit, accessed on 18 May 2021.

Due to the large selection of binary similarity metrics, the average values of the different metrics can be used for the comparison of the assessors, as the simplest consensus option. The reason for this choice is that we cannot arbitrarily select the best similarity metric a priori. By contrast, the merit of consensus metrics/methodologies have been extensively demonstrated [16,17,18]. Briefly, using the average of the 44 similarity metrics is supported by the maximum likelihood principle, which “yields a choice of the estimator as the value for the parameter that makes the observed data most probable” [19]. In effect, this means that all of the individual similarity metrics express the true (unknown) similarities with some errors (biases and random errors as well), but computing their average cancels out these errors, at least partially. Thus, average similarity metrics were calculated for each pair of samples in each dataset and for every assessor. Those assessors who produce smaller similarities on average (between samples) can be considered to be more sensitive to differences between the tested samples. 

#### 2.2.2. Check-All-That-Apply Data Evaluation

The data matrix obtained after running and averaging the 44 similarity measures on the binary CATA data table consists of columns corresponding to the assessors, and rows corresponding to the pairs of products. The values in the matrix express the average similarity metrics computed for the given assessor and the given pair of samples. These pair-wise differences were subjected to a (column-wise) agglomerative hierarchical cluster analysis (AHC) using Ward’s method and the Euclidean distance to create two clusters, one representing assessors with good discrimination ability (e.g., lower average similarity values) and the other containing assessors with poor discrimination ability (e.g., higher average similarity values). In order to visualize the differences between the two groups, correspondence analysis (CA) was computed for the total panel and for the created two clusters (good/poor discrimination ability). AHC was run on the matrix of similarity measures while CA was run on the binary data matrix obtained from the sensory tests. Optimal cluster numbers were determined using the Silhouette index [20]. AHC and CA were calculated using XL-Stat ver. 2019.2.2 [21].

## 3. Results and Discussion

**Figure 1:**
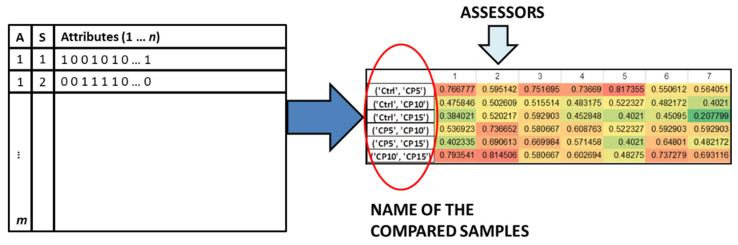
The workflow of the calculation of similarity metrics from CATA experiments. Assessors are marked with “A” and Samples are marked with “S” in the left side of the plot, with “n” being the number of attributes and “m” the number of assessors.

The 44 similarity metrics were calculated for each dataset in the following way: the original binary dataset contained the attributes in the columns and the samples in the rows. The assessors were also assigned to the rows. After the calculation of the 44 metrics, the averaged values for each sample combination can be found in the cells of the matrix, where the assessors were in the columns and the compared sample combinations in the rows. The workflow can be followed in Figure 1.

**Figure 2:**
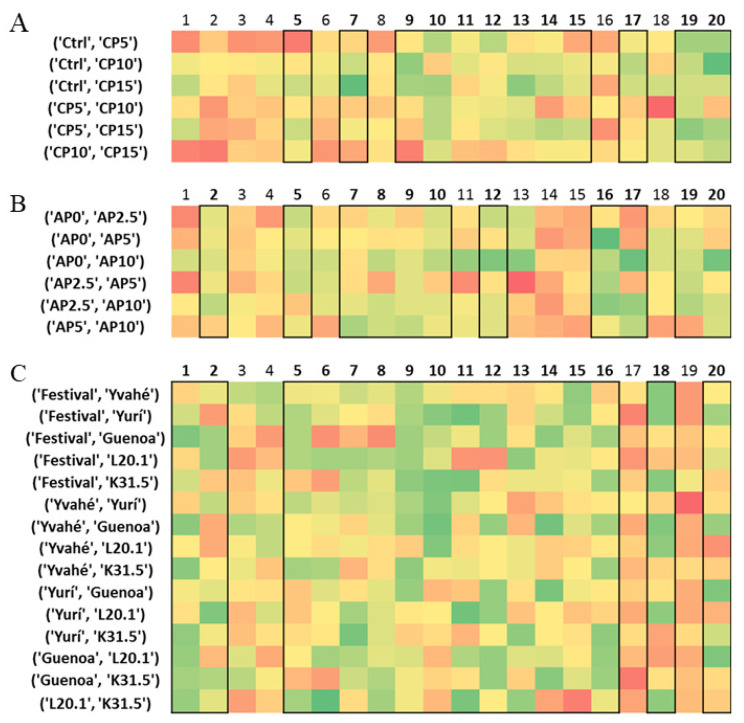
Excerpts from the three heatmaps for the three case studies (**A**: Cricket, **B**: Apple pomace enriched and **C**: Strawberry). Red color means lower, while green color means better discrimination ability. Sample names are in the first columns, while participants are denoted by numbers.

The average similarity values for each assessor are plotted in a heatmap visualization, colored dark green to red, from the lowest similarity to the highest similarity. To allow for easier discrimination among the assessors, averages were calculated from the similarity values column-wise (assessor average), as well as for the whole data matrix (grand average). Those assessors, whose assessor averages are below the grand average (defined here as a consensus limit) can be considered as the better ones, i.e., having higher-than-average discrimination ability. These individuals were marked with brackets in Figure 2. Figure 2 presents a part of the results of the three different datasets, the full heatmaps can be found in the supplementary material (Figures S1–S3). The grand averages (or consensus limits), the number of the selected assessors based on the limit value, and the row maximum and minimum values are reported in Table 3. The color ranges of the heatmaps were specified by the latter two parameters.

### 3.1. Cricket Enriched Biscuit Dataset

Based on the average similarity measures of the assessors, 37 participants were marked as good discriminators (highlighted with black boxes on Figure 2). To provide an even finer distinction and grouping of the assessors (based on the discrimination of the samples pair-wise), agglomerative hierarchical cluster analysis (AHC) was used. 

**Figure 3:**
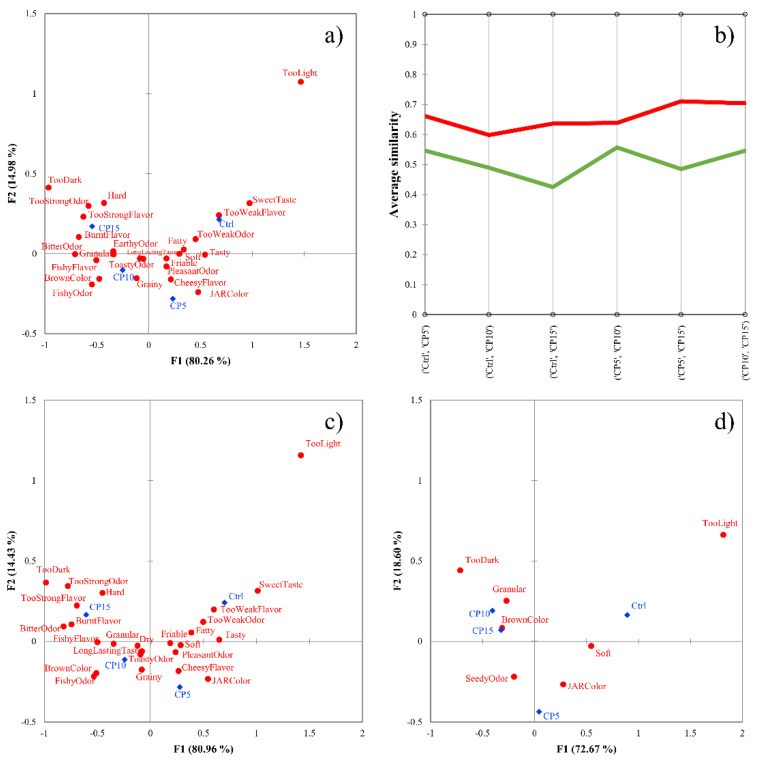
(**a**–**d**) Results of the cricket-enriched biscuit dataset. Correspondence analysis of the total sample (n = 67). (**a**), profile plot of the agglomerative hierarchical cluster analysis with Euclidean distance and Ward’s method (**b**), correspondence analysis of good discriminators (n = 56) (**c**), correspondence analysis of poor discriminators (n = 11) (**d**). Products are marked with blue dots, attributes are marked with red squares in panels (**a**,**c**,**d**), cluster of good discriminators is colored by green, while poor discriminators are colored by red in panel (**b**). Samples contained 0% (Ctrl), 5% (CP5), 10% (CP10), and 15% (CP15) cricket powder.

In order to visualize the effects of the grouping based on the similarity values, correspondence analysis was carried out on the total panel data (Figure 3a). Figure 3a presents only the significant check-all-that-apply (CATA) attributes determined by Cochran’s Q test. The total inertia shows high explained variance (95.24%) and the four products are placed into four different quadrants. Additionally, the products are arranged in a “U” shape from left to right based on the amount of added cricket powder (from Ctrl (0%) to CP15 (15%)). The detailed analysis of the product relationship among products and attributes will be published elsewhere. 

AHC was applied to the pair-wise sample average similarity values of the assessors, as presented by Figure 1. Silhouette clustering indices were computed to validate the cluster numbers. The highest average Silhouette index was found in the case of two clusters (0.1810), hence this will be used later. 

Figure 3b presents the profile plot of AHC, showing two clearly distinguishable clusters. In the plot, lower average similarity values mean better discrimination, while values closer to 1 mean poor discrimination between samples. The lowest average similarity values were obtained in the case of Ctrl vs. CP15, which is expected since these are the two most different products. The profile plot shows that cluster 1 (green line) showed better discrimination among products, creating the group of good discriminators. Altogether 56 consumers were identified as good discriminators and 11 as poor ones. 

Figure 3c,d presents the cluster analysis (CA) of the two clusters separately. The number of significant CATA terms (Table 1) was higher for the good discriminators than for the total panel (19 vs. 17) and lower for the poor discriminators (7). Additionally, while Ctrl and CP5 were discriminated well, the difference between CP10 and CP15 is less pronounced based on the total panel and the good discriminators.

### 3.2. Apple Pomace-Enriched Biscuit Dataset

**Figure 4:**
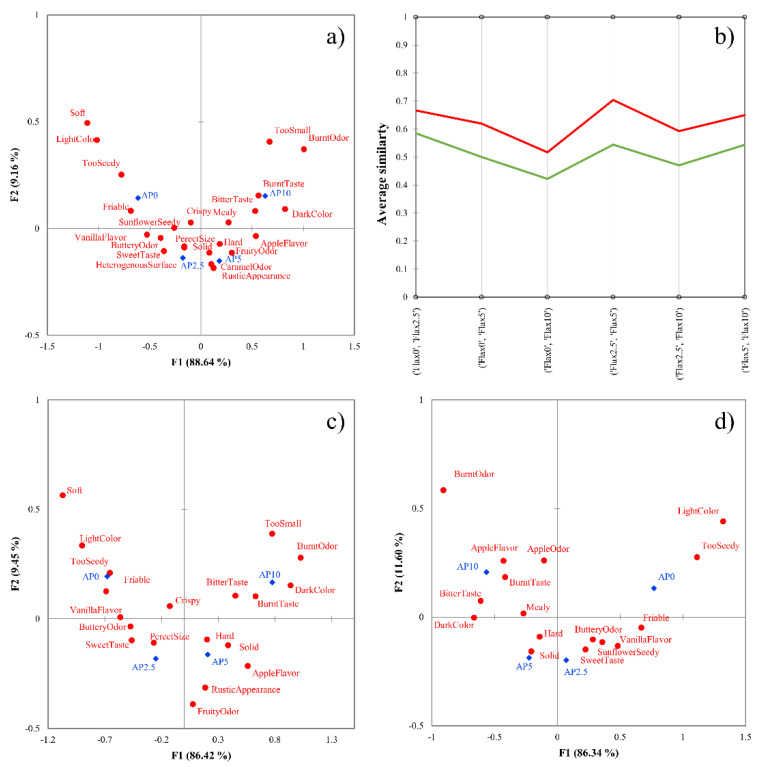
(**a**–**d**) Results of the apple pomace-enriched biscuit dataset. Correspondence analysis of the total sample (n = 60). (**a**), profile plot of the agglomerative hierarchical cluster analysis with Euclidean distance and Ward’s method (**b**), correspondence analysis of good discriminators (n = 37) (**c**), correspondence analysis of poor discriminators (n = 23) (**d**). Products are marked with blue dots, attributes are marked with red squares in panels (**a**,**c**,**d**), cluster of good discriminators is colored by green, while poor discriminators are colored by red in panel (**b**). Samples contained 0% (AP0), 2.5% (AP2.5), 5% (AP5), and 10% (AP10) ground apple pomace.

Results of the apple pomace-enriched biscuit dataset are presented in Figure 4. Figure 4a shows the CA results on the total panel data, which presents a “U” shape according to the amount of added apple pomace to the products. AHC of the binary similarity metrics identifies two distinct clusters (average Silhouette width: 0.2145) (Figure 4b). The first cluster shows lower similarity values, meaning they discriminate the products better. Figure 4c presents the CA of cluster 1 (good discriminators), which showed better discrimination between the product pairs, while Figure 4d shows the results of cluster 2. CA shows a higher number of significant attributes compared to Figure 4a, and the products are also better differentiated.

### 3.3. Strawberry Dataset

**Figure 5:**
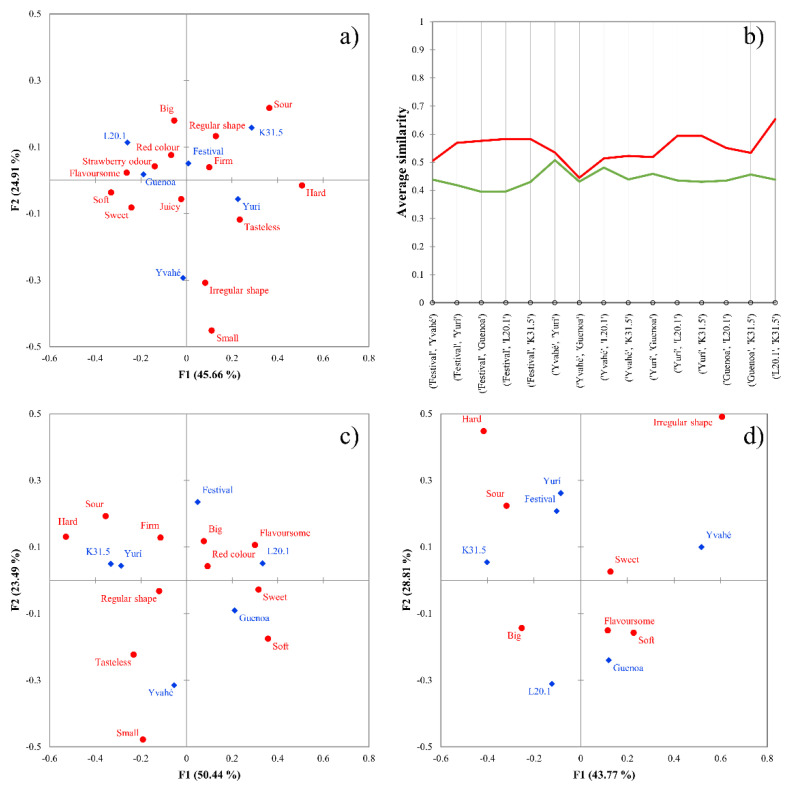
(**a**–**d**) Results of the strawberry dataset. Correspondence analysis of the total sample (n = 117). (**a**), profile plot of the agglomerative hierarchical cluster analysis with Euclidean distance and Ward’s method (**b**), correspondence analysis of good discriminators (n = 81) (**c**), correspondence analysis of poor discriminators (n = 35) (**d**). Varieties are marked with blue dots, attributes are marked with red squares in panels (**a**,**c**,**d**), cluster of good discriminators is colored by green, while poor discriminators are colored by red in panel (**b**).

Analysis of total panel results are similar to those published by other authors evaluating the same data set [1,8,22], namely that the products were described by the CATA terms (Figure 5a). 

Analyzing the optimal cluster numbers, Silhouette index revealed that two clusters would be optimal (average Silhouette width: 0.1138), which is similar to those cluster numbers found in the previous case study. Figure 5b presents the profile plot of the two clusters, where again, the two clusters can be described as good and poor discriminators (Figure 5c,d). Cluster 1 (good discriminators) shows average similarity values around 0.4–0.5, while cluster 2 (poor discriminators) shows slightly higher values (0.5–0.6). It is important to note that some samples showed higher difference. For example, the first five sample pairs in Figure 5b, while others (located in the middle of the plot) showed very similar results, thus no difference between the two clusters. This might be due to the high similarity of the samples. The strawberry dataset consisted of six varieties, which greatly raises the number of combinations during a pair-wise comparison. (Compared to the cricket powder enriched biscuit dataset, where six combinations were calculated, here 15 pairs can be enumerated.)

Compared to the previous dataset, the average similarity measure values are lower, meaning that the participants in the strawberry group discriminated the samples better compared to the group evaluating the cricket powder enriched biscuit samples. Considering this observation, we propose that the similarity measures could be generalized to compute a global measure describing the discriminator power of the test. However, it should be noted that this is a combined result, we cannot judge the participants (e.g., their discriminatory power) independently of the similarities of the products.

### 3.4. Comparison of Product Liking

Comparison of overall liking values between the two consumer groups determined by agglomerative hierarchical clustering of the similarity values is presented by Table 4. In the case of cricket enriched oat biscuits and strawberry varieties, the cluster showing lower similarity values, e.g., the one having better discrimination ability, shows significant differences between the samples based on overall liking. In the case of cricket enriched oat biscuit data set, Ctrl and CP5 is significantly more liked compared to the products containing higher amounts of cricket powder. Although a similar tendency can be seen in the case of C2, there is no statistically justifiable difference between the OAL of the samples. C2 consisted of participants who liked the products similarly and did not make any difference among them. 

The strawberry dataset shows also that members of C1 differentiated the products on a higher level, their mean OAL across products ranged between 4.358 (Yurí) and 5.753 (L20.1) and significant differences were found. Interestingly, while the OAL values reported by the members of C2 span across a slightly greater range (5.0–6.6), due to the wide distribution (i.e., large variance) of the values, the differences between the products are statistically not significant.

The apple pomace-enriched biscuits dataset showed a different result. In this case, both clusters showed significant differences among samples with the same pattern, meaning that AP0 proved to be the most liked and AP10 as the least liked one. It has to be mentioned that these products were highly different because the added amount of apple pomace changed the texture, color, and taste to a great extent, which is why the consumers differentiated the products very well. 

### 3.5. Comparison with Panelist Agreement

Panelist agreement was calculated by the CATATIS method introduced recently [9]. CATATIS calculates the so-called homogeneity index, describing the global agreement among the respondents. The homogeneity index was found to be satisfactory for the cricket-enriched biscuit dataset (42.6%), while the strawberry dataset showed lower homogeneity (37.2%). The highest homogeneity index was achieved for the apple pomace-enriched biscuits (52.1%), showing that the assessors had similar opinions about the products. A discrimination index was calculated to characterize the whole dataset by computing the grand average of the binary similarity measures of each assessor. The Best discrimination was obtained in the case of the strawberry dataset (47.3%), while the cricket-enriched and apple pomace-enriched biscuit datasets showed poor discrimination (53.3% and 55.5%, respectively). It should be noted that unlike the homogeneity index, the lower discrimination index denotes better discrimination.

**Figure 6:**
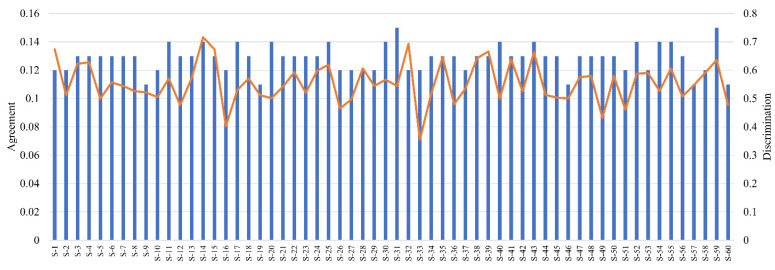
Agreement and discrimination ability of assessors from the flax biscuit case study. Agreement is presented by bars, while discrimination ability is presented by a line.

The authors would emphasize the joint application of the homogeneity index (as the measure of panelist agreement) and the introduced methodology (as the measure of discrimination ability) in order to identify assessors with good or weak performance. An example of this joint application is presented by Figure 6, where the agreement and discrimination ability of assessors from the apple pomace-enriched biscuit case study are plotted. The figure presents that there is no correlation between the two metrics, i.e., a panelist having good agreement with the panel does not guarantee that he/she discriminates the samples well. The proposed joint application gives detailed information about the dataset and the panelist’s performance as well, by applying different filtering methods, e.g., the one proposed by Llobell, Cariou, et al. (2019). 

## 4. Conclusions

The proposed methodology aims to fill a gap in check-all-that-apply (CATA) tests by providing a method to assess the discrimination ability of the panelists participating in CATA evaluations. The method fits into the scope of newly introduced CATA data analysis methods. The proposed analysis is easy to run using our open-source python package, available at https://github.com/davidbajusz/fpkit, accessed on 18 May 2021. The presented results of the three case studies show that a simple clustering of the computed binary similarity measures results in higher quality correspondence analysis with more significant attributes, as well as better sample discrimination (even according to overall liking).

It must be mentioned that good discrimination does not mean good repeatability and/or panel agreement. In order to describe the performance of a panel/panelist correctly, all three metrics should be calculated. In the present paper, the joint application of discrimination ability and repeatability has been introduced, however by conducting replicate sessions (which is more common with trained panels), repeatability can also be calculated using 2 × 2 tables [23].

The distance metric and linkage rule were defined as the most popular ones, namely Euclidean distance and Ward’s method. Further analysis could assess the effect of the combination of distance metrics and linkage rules on the separation of good and poor discriminators, ideally on a greater number of datasets.

In particular, since the sensory similarity of the products is difficult to assess prior to the tests, the method should be tested with a wide range of products, from highly similar to highly different. Higher average similarity values (e.g., poor discrimination) might be achieved due to the high similarity of the samples and not only because of the panelist’s poor performance.

Our proposed method gives a new tool to the hands of consumer sensory panel leaders for the assessment of the discrimination ability of consumers participating in CATA tests. As the used scripts are free to download and use, these calculations can be built into any data analysis routines easily. The obtained information improves the reliability of CATA tests, as well as gives another piece of important information regarding the performance of consumer panels. Application of binary similarity measures with the earlier proposed homogeneity index gives a complete evaluation of the consumer panels.

A further future direction would be the application of the proposed method on trained panel data, where the panel agreement and discrimination ability are more important than they are during a consumer test. Additionally, comparison of binary similarity metrics to the existing methods used to assess the discrimination ability would further validate the proposed approach in trained panel tests. A further advantage of the proposed approach would be to use as a screening method for the identification of well-performing consumers who could be potentially transferred into trained panels after completing the required trainings.

## Figures and Tables

**Table 1:** Attributes evaluated by consumers using CATA questions for sensory characterization of different product categories in the four studies included in this research.

Study	CATA Terms
Cricket enriched biscuit	too dark, too light, nice color, brown color, grainy, too strong odor, too weak odor, cheesy odor, bitter odor, seedy odor, earthy odor, sunflower-seedy odor, toasty odor, pleasant odor, fishy odor, friable, hard, soft, crumbly, fatty, crispy, granular, dry, too strong flavor, too weak flavor, cheesy flavor, seedy flavor, spicy flavor, salty taste, sunflower-seedy flavor, toasty flavor, tasty, sweet taste, sticky, piquant, fishy flavor, burnt flavor, long lasting taste
Apple pomace biscuit enriched biscuit	light, dark, homogeneous, heterogenous, seedy, rustic, perfect size, small, fruity odor, citrus odor, apple odor, buttery odor, caramel odor, burnt odor, coconut odor, hard, flexible, chewy, crispy, solid, mealy, sunflower seedy, soft, sticky, friable, tasteless, vanilla flavor, fruity flavor, citrus flavor, apple flavor, caramel flavor, sweet bitter, sour, burnt
Strawberry	sweet, sour, strawberry flavor, strawberry odor, flavorsome, tasteless, red color, irregular shape, regular shape, small, big, firm, hard, soft, juicy, dry

**Table 2:** 2 × 2 table for the calculation of the binary similarity metrics.

p = a + b + c + d	Sample 2	
Sample 1	1 (Attribute present)	0 (Attribute absent)
1 (Attribute present)	a	b
0 (Attribute absent)	c	d

**Table 3:** Summary of the average similarity values and row minimum and maximum values for the heatmaps in each case study.

	Consensus Limit	Selected Assessors *	Row Minimum	Row Maximum
Dataset 1 (cricket)	**0.53**	**37/67**	**0.21**	**0.89**
Dataset 2 (apple pomace enriched biscuit)	**0.55**	**32/60**	**0.10**	**0.96**
Dataset 3 (strawberry)	**0.47**	**63/117**	**0.15**	**0.89**

* Selected assessors, whose assessor averages are below the grand average (defined here as a consensus limit) can be considered as the better ones, i.e., having higher-than-average discrimination ability.

**Table 4:** Analysis of variance and Tukey post-hoc tests of overall liking variable conducted on the two clusters separately for all three product groups. Letters indicate homogeneous subgroups determined by Tukey post hoc test. C1 indicates the cluster achieving lower average similarity values, e.g., having better discrimination ability.

**Cricket**	**C1**		**C2**
Ctrl	6.536 ^b^	CP5	6.818 ^a^
CP5	6.339 ^b^	Ctrl	6.727 ^a^
CP10	5.357 ^a^	CP10	6.182 ^a^
CP15	4.518 ^a^	CP15	6.091 ^a^
**Apple pomace enriched**	**C1**		**C2**
AP0	6.216 ^c^	AP0	6.304 ^c^
AP2.5	5.541 ^bc^	AP2.5	5.435 ^bc^
AP5	5.081 ^b^	AP5	4.870 ^ab^
AP10	3.568 ^a^	AP10	3.652 ^a^
**Strawberry**	**C1**		**C2**
L20.1	5.753 ^b^	L20.1	6.571 ^a^
Festival	5.247 ^ab^	Guenoa	6.171 ^a^
Guenoa	5.136 ^ab^	Festival	6.029 ^a^
Yvahé	4.938 ^ab^	Yvahé	5.486 ^a^
K31.5	4.469 ^a^	K31.5	5.229 ^a^
Yurí	4.358 ^a^	Yurí	4.971 ^a^

Letters denote homogenous subgroups determined by Tukey post-hoc tests.

## Data Availability

The data presented in this study are available on request from the corresponding author. The data are not publicly available due to legal restrictions.

## Supplementary Materials

The following are available online at https://www.mdpi.com/article/10.3390/foods10051123/s1, Figure S1: The heatmap for Cricket, Figure S2: The heatmap for Apple pomace enriched. Figure S3: The heatmap for Strawberry.

## References

[1] Meyners M., Castura J.C., Varela P., Ares G. (2014). Check-All-That-Apply Questions. Novel Techniques in Sensory Characterization and Consumer Profiling.

[2] Alexi N., Nanou E., Lazo O., Guerrero L., Grigorakis K., Byrne D.V. (2018). Check-All-That-Apply (CATA) with semi-trained assessors: Sensory profiles closer to descriptive analysis or consumer elicited data?. Food Qual. Prefer..

[3] Jaeger S.R., Beresford M.K., Paisley A.G., Antúnez L., Vidal L., Cadena R.S., Giménez A., Ares G. (2015). Check-all-that-apply (CATA) questions for sensory product characterization by consumers: Investigations into the number of terms used in CATA questions. Food Qual. Prefer..

[4] Meyners M., Castura J.C., Carr B.T. (2013). Existing and new approaches for the analysis of CATA data. Food Qual. Prefer..

[5] Plaehn D. (2012). CATA penalty/reward. Food Qual. Prefer..

[6] Ares G., Dauber C., Fernández E., Giménez A., Varela P. (2014). Penalty analysis based on CATA questions to identify drivers of liking and directions for product reformulation. Food Qual. Prefer..

[7] Jaeger S.R., Chheang S.L., Yin J., Bava C.M., Gimenez A., Vidal L., Ares G. (2013). Check-all-that-apply (CATA) responses elicited by consumers: Within-assessor reproducibility and stability of sensory product characterizations. Food Qual. Prefer..

[8] Llobell F., Cariou V., Vigneau E., Labenne A., Qannari E.M. (2019). A new approach for the analysis of data and the clustering of subjects in a CATA experiment. Food Qual. Prefer..

[9] Llobell F., Giacalone D., Labenne A., Qannari E.M. (2019). Assessment of the agreement and cluster analysis of the respondents in a CATA experiment. Food Qual. Prefer..

[10] Næs T., Brockhoff P.B., Tomic O. (2010). Statistics for Sensory and Consumer Science.

[11] Peltier C., Brockhoff P.B., Visalli M., Schlich P. (2014). The MAM-CAP table: A new tool for monitoring panel performances. Food Qual. Prefer..

[12] Bajusz D., Rácz A., Héberger K. (2015). Why is Tanimoto index an appropriate choice for fingerprint-based similarity calculations?. J. Cheminform..

[13] Rácz A., Bajusz D., Héberger K. (2018). Life beyond the Tanimoto coefficient: Similarity measures for interaction fingerprints. J. Cheminform..

[14] Rácz A., Andrić F., Bajusz D., Héberger K. (2018). Binary similarity measures for fingerprint analysis of qualitative metabolomic profiles. Metabolomics.

[15] Todeschini R., Consonni V., Xiang H., Holliday J., Buscema M., Willett P. (2012). Similarity Coefficients for Binary Chemoinformatics Data: Overview and Extended Comparison Using Simulated and Real Data Sets. J. Chem. Inf. Model..

[16] Héberger K., Kollár-Hunek K. (2011). Sum of ranking differences for method discrimination and its validation: Comparison of ranks with random numbers. J. Chemom..

[17] Sziklai B.R., Héberger K. (2020). Apportionment and districting by Sum of Ranking Differences. PLoS ONE.

[18] Lagares L.M., Minovski N., Alfonso A.Y.C., Benfenati E., Wellens S., Culot M., Gosselet F., Novič M. (2020). Homology modeling of the human p-glycoprotein (Abcb1) and insights into ligand binding through molecular docking studies. Int. J. Mol. Sci..

[19] Hastie T., Tibshirani R., Friedman J. (2001). Overview of supervised learning. Elements of Statistical Learning, Data Mining, Inference, Prediction.

[20] Rousseeuw P.J. (1987). Silhouettes: A graphical aid to the interpretation and validation of cluster analysis. J. Comput. Appl. Math..

[21] (2019). Addinsoft XLSTAT Statistical and Data Analysis Solution. New York, USA. https://www.xlstat.com.

[22] Ares G., Jaeger S.R. (2013). Check-all-that-apply questions: Influence of attribute order on sensory product characterization. Food Qual. Prefer..

[23] Meyners M., Castura J.C., Worch T. (2016). Statistical evaluation of panel repeatability in Check-All-That-Apply questions. Food Qual. Prefer..

