# Supplementary figures and images for: Discrimination Ability of Assessors in Check-All-That-Apply Tests: Method and Product Development
Source: Foods. 2021 May 19;10(5):1123. doi: 10.3390/foods10051123 (PMC8158734; doi:10.3390/foods10051123)

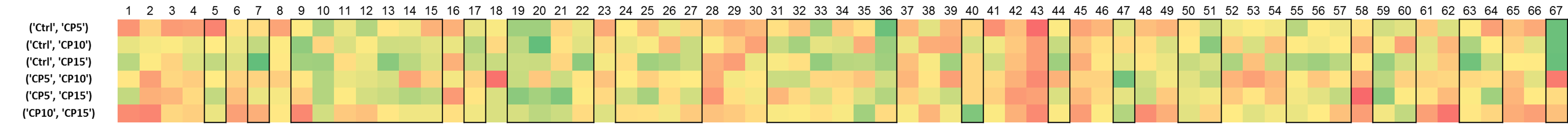

Supplement: Supplementary file 1 [file foods-10-01123-s001.zip › SupplementaryMaterial/FigureS1.tif]

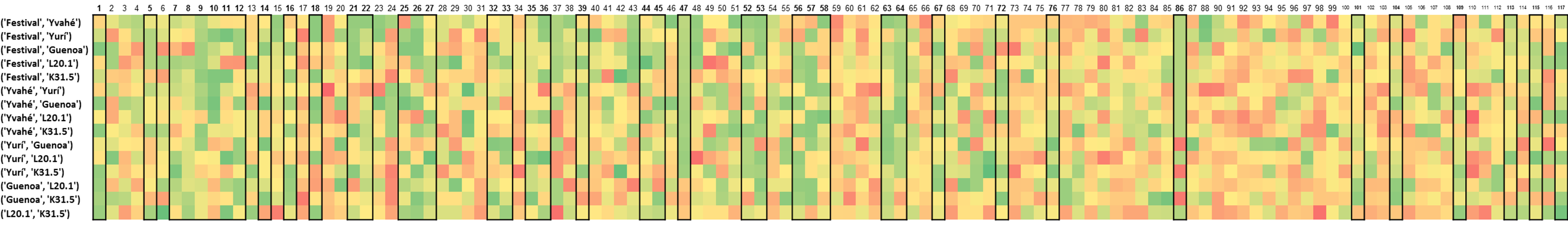

Supplement: Supplementary file 1 [file foods-10-01123-s001.zip › SupplementaryMaterial/FigureS2.tif]

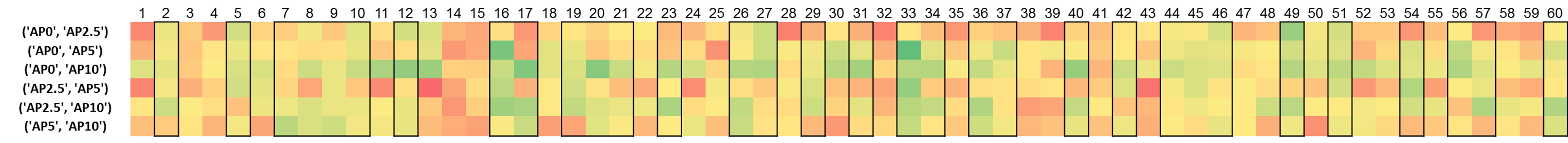

Supplement: Supplementary file 1 [file foods-10-01123-s001.zip › SupplementaryMaterial/FigureS3.tif]
